# Anti-Bacterial and Microecosystem-Regulating Effects of Dental Implant Coated with Dimethylaminododecyl Methacrylate

**DOI:** 10.3390/molecules22112013

**Published:** 2017-11-20

**Authors:** Bolei Li, Yang Ge, Yao Wu, Jing Chen, Hockin H. K. Xu, Minggang Yang, Mingyun Li, Biao Ren, Mingye Feng, Michael D. Weir, Xian Peng, Lei Cheng, Xuedong Zhou

**Affiliations:** 1 State Key Laboratory of Oral Diseases, Sichuan University, Chengdu 610041, China; bolei.d.li@gmail.com (B.L.); yang_ge_dentist@hotmail.com (Y.G.); sallychen.jc@gmail.com (J.C.); limingyun@scu.edu.cn (M.L.); renbiao@scu.edu.cn (B.R.); fengmingye@gmail.com (M.F.); 2 National Clinical Research Center for Oral Diseases, Sichuan University, Chengdu 610041, China; 3 Department of Cariology and Endodontics, West China School of Stomatology, Sichuan University, Chengdu 610041, China; 4 National Engineering Research Center for Biomaterials, Sichuan University, Chengdu 610064, China; wuyao@scu.edu.cn (Y.W.); minggang_yang@163.com (M.Y.); 5 Department of Advanced Oral Sciences & Therapeutics, University of Maryland Dental School, Baltimore, MD 21201, USA; Hxu@umaryland.edu (H.H.K.X.); MWeir@umaryland.edu (M.D.W.)

**Keywords:** dental implant, peri-implant diseases, dimethylaminododecyl methacrylate, antimocrobial material, saliva-derived biofilm

## Abstract

The effects of dimethylaminododecyl methacrylate (DMADDM) modified titanium implants on bacterial activity and microbial ecosystem of saliva-derived biofilm were investigated for the first time. Titanium discs were coated with DMADDM solutions at mass fractions of 0 mg/mL (control), 1, 5 and 10 mg/mL, respectively. Biomass accumulation and metabolic activity of biofilms were tested using crystal violet assay and MTT (3-(4,5-Dimethyl-thiazol-2-yl)-2,5-diphenyltetrazolium bromide) assay. 16S rRNA gene sequencing was performed to measure the microbial community. Live/dead staining and scanning electron microscopy (SEM) were used to value the structure of biofilm. The results showed that the higher mass fraction of DMADDM the coating solution had, the significantly lower the values of metabolic activity and accumulated biofilms got, as well as fewer live cells and less extracellular matrix. Moreover, 5 mg/mL of DMADDM was the most effective concentration, as well as 10 mg/mL. In microecosystem-regulation, the DMADDM modified titanium implant decreased the relative abundance of *Neisseria* and *Actinomyces* and increased the relative abundance of *Lactobacillus*, a probiotic for peri-implant diseases. In conclusion, via inhibiting growth and regulating microecosystem of biofilm, this novel titanium implant coating with DMADDM was promising in preventing peri-implant disease in an ‘ecological manner’.

## 1. Introduction

As a widely accepted restorative, dental implants have an initial success rate of up to 98% [1]. However, five years later, the success rate ranges between 90.1% and 95.4%, and after 10 and 16 years it reduces to about 89% and 83%, respectively [2]. Local infection and lack of sufficient osseointegration contributed the most to complications. Hence, two main implant-related infectious diseases: peri-implantitis and peri-implant mucositis emerged in the past three decades. Evidence reveals that 50% to 90% of implants suffer peri-implant mucositis, while 20% of implants develop peri-implantitis with an average function time of 5 to 11 years [3]. Microbial biofilm accumulation is the major cause for implant-related infections and inflammatory responses [4,5]. In addition, more and more evidence shows that the micro-ecosystem dysbiosis of biofilms is also tightly associated with peri-implant diseases [6,7,8,9,10].

Therefore, infection-free establishment of bone-implant integration has become a persistent challenge in oral rehabilitation. Various efforts have been made to enhance the anti-bacteria ability of dental implants to resist peri-implant diseases [11,12,13,14,15,16,17]. Dimethylaminododecyl methacrylate (DMADDM), a novel quaternary ammonium salts (QAS), is known as a long-lasting and remarkable antibacterial additive with good biocompatibility, which had been incorporated into different dental materials [18,19,20,21,22]. Besides, DMADDM incorporated in dental materials is able to reshape the microbial structure of biofilm to the healthy condition to enhance the anti-caries effect [23,24,25].

Recently, Yang et al. [26] immobilized DMADDM onto alkali-heat-treated titanium (AH-Ti) discs benefiting from poly dopamine (PDA) hydrochloride with suitable biocompatibility and increasing roughness. The anti-bacteria ability of these titanium discs was verified using three-species biofilms in vitro [14]. Beyond the pathogenic bacteria, the anti-bacteria effect on microbial population is not clear yet, which is composed of tens to hundreds of oral bacterial species [7,8,9,10]. Moreover, the microecosystem-regulating effect of DMADDM modified dental implant has not been studied, although peri-implant disease was associated with microecosystem dysbiosis of biofilms [6,7,8,9,10].

Therefore, the aims of this study were to study the anti-bacteria and microecosystem-regulating effects of DMADDM modified dental implants using a saliva-derived biofilm model in vitro. It was hypothesized that: (1) DMADDM was able to inhibit the growth and metabiotic activity of saliva-derived bacteria; (2) DMADDM could regulate the microbial ecosystem of saliva-derived biofilm to more healthy condition.

## 2. Results

### 2.1. Biomass Accumulation and Metabolic Activity

**Figure 1:**
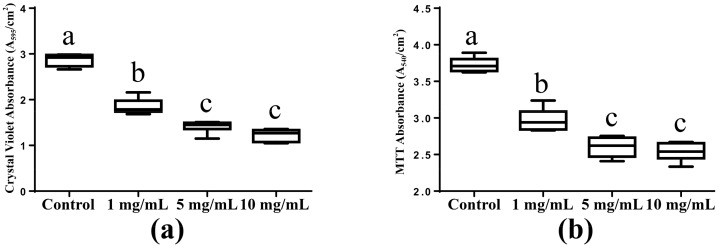
Biomass accumulation and metabolic activity of saliva-derived biofilms on titanium discs. (**a**) Crystal violet assay for the biomass accumulation of saliva-derived biofilms on the titanium discs. (*n* = 6); (**b**) MTT assay for the metabolic activity of the biofilms (*n* = 6). Significant difference occurs between the bars marked with the different letters (a, b, c) (*p* < 0.05).

The biomass accumulation of the saliva-derived biofilms was measured using crystal violet assay, which was presented in Figure 1a. Compared with the control group (no DMADDM), the biomass accumulation of biofilms on the DMADDM modified titanium disc decreased significantly (*p* < 0.05). Less biomass accumulation was found in 5 mg/mL group (5 mg/mL DMADDM in coating solution) and 10 mg/mL group (10 mg/mL DMADDM in coating solution) significantly (*p* < 0.05), compared with 1 mg/mL group (1 mg/mL DMADDM in coating solution). No significant difference between 5 mg/mL group and 10 mg/mL group was found.

The metabolic activity of the saliva-derived biofilms was revealed in Figure 1b. The metabolic activity decreased from control group and 1 mg/mL group to the 5 mg/mL and 10 mg/mL groups significantly (*p* < 0.05). Compared with the control group, metabolic activity of 1 mg/mL group decreased significantly (*p* < 0.05). In addition, the biofilms on the titanium discs coated with 5 mg/mL and 10 mg/mL DMADDM solution showed the same metabolic activity statistically.

### 2.2. Live/Dead Bacteria Staining

**Figure 2:**
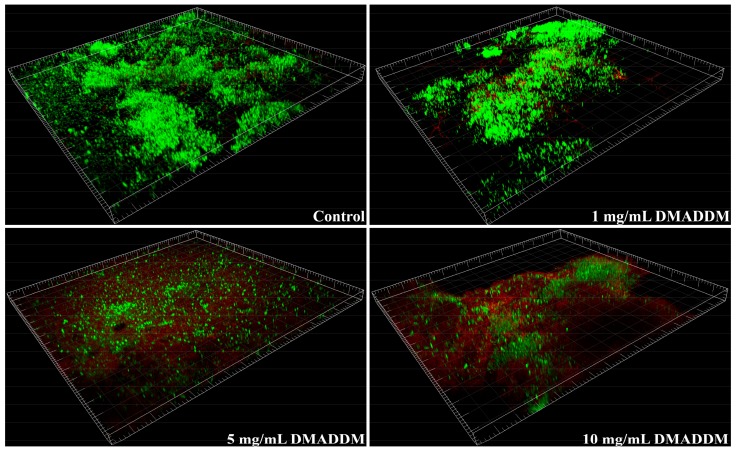
Confocal laser scanning microscope (CLSM) of saliva-derived biofilms. Live/dead staining of biofilms on the titanium discs of the four groups. Live cells were stained green, and dead cells were stained red.

The result of the live/dead bacteria staining was exhibited in Figure 2. More live cells could be detected in the biofilms from control group, rather than the biofilms on DMADDM modified titanium discs. Dead cells were scarcely detected. Compared with the biofilm from 1 mg/mL group, those from 5 mg/mL and 10 mg/mL groups contained more dead cells. Most of the cells in biofilms from 5 mg/mL and 10 mg/mL groups were killed. The results indicated that the higher mass fraction of DMADDM the coating solution had, the higher the rate of dead cells in biofilms was.

### 2.3. The Microbial Community of Saliva-Derived Biofilms

**Figure 3:**
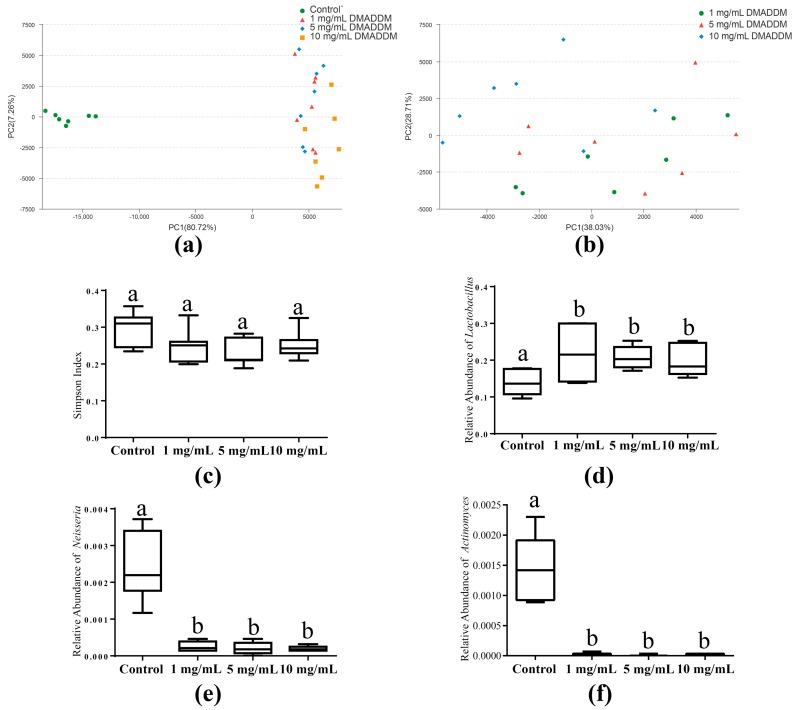
The microbial community of saliva-derived biofilms. (**a**) Principal component analysis (PCA) score plot of control group (green), 1 mg/mL DMADDM group (red) 5 mg/mL DMADDM group (blue) and 10 mg/mL DMADDM group (yellow); (**b**) PCA score plot of 1 mg/mL DMADDM group (green) 5 mg/mL DMADDM group (yellow) and 10 mg/mL DMADDM group (blue); (**c**) Alpha diversity of every group was measured with the Simpson index; (**d**–**f**) showed the relative abundance of *Lactobacillus*, *Neisseria*, and *Actinomyces*, respectively. Significant difference can be seen between the bars marked with different letters (a, b) (*p* < 0.05).

Principal component analysis (PCA) analysis showed that the biofilms of control group separated from those of 1, 5 and 10 mg/mL groups distinctly (Figure 3a). While the biofilms on DMADDM modified titanium discs clustered together and hardly separated from each other (Figure 3b). Alpha diversity did not contribute to causing the difference in microbial community between control group and the others, because no significant difference (*p* > 0.05) between them was found in the Simpson index (Figure 3c). However, the microbial composition of the control group was different from those of DMADDM modified group. Higher abundance of *Lactobacillus* (*p* < 0.05) was detected in the DMADDM modified group (Figure 3d). In contrast, the increasing abundance of *Neisseria* and *Actinomyces* (*p* < 0.05) could be found in control group (Figure 3e,f). Besides, the relative abundance of *Porphyromonas* presented no significant difference (*p* > 0.05) among the four groups (Figure S1).

### 2.4. Scanning Electron Microscopy (SEM) Observation

**Figure 4:**
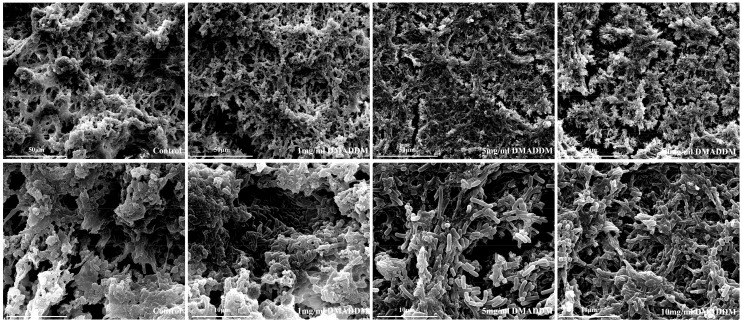
Scanning electron microscopy (SEM) micrographs of saliva-derived biofilms on titanium discs.

Figure 4 showed the SEM micrographs of the biofilms on titanium discs. More extracellular matrix could be found in the biofilms from control group, compared with that on DMADDM modified titanium discs, and the bacteria were wrapped with the extracellular matrix. The biofilm matrix of 1 mg/mL group was less than that of control group, but more than that of 5 mg/mL and 10 mg/mL group. Part of the bacteria were exposed from the extracellular matrix, and little more bacillus could be detected. In 5 mg/mL and 10 mg/mL groups, most of the bacteria were free of the extracellular matrix, bacillus could be found clearly.

## 3. Discussion

In previous studies [12,13,14,15], controlled multi-species biofilm models were usually used to investigate the anti-peri-implantitis effect of modified dental implants in vitro. However, these controlled multi-species biofilms were composed of pathogenic bacteria, without the beneficial bacteria that play an important role in contributing a healthy balance of microbiota. Thus, these models have their limitations in testing the effect of modified dental implant on microbial ecosystem of biofilms. In the present study, in order to study the anti-bacteria effect on microbial population and microecosystem-regulating effect of DMADDM modified titanium implant, the saliva-derived biofilms cultured with SHI medium were chosen, which had been confirmed as being able to support a diversified oral microbial community in vitro [27,28]. The proteins derived from blood and saliva, being considered to provide additional bacterial adhesion site [29,30,31], were compositions of SHI medium [27] and saliva. They made the saliva-derived biofilms in vitro more similar to those in vivo. In addition, to provide more comprehensive and accurate fingerprints of microbial community of saliva-derived biofilm, we used 16S rRNA gene sequencing technique in the present study, instead of the denaturing gradient gel electrophoresis (DGGE) which was widely used previously [11,27,32,33,34,35].

In traditional opinion, QAS has a ‘contact killing’ antibacterial mechanism, as the QAS coating materials’ surfaces are highly positively charged (*N*+). When it comes into contact with the negatively charged bacteria, long fatty alkyl chains of QAS can penetrate the bacteria membrane causing cytoplasmic leakage [36,37]. Our results revealed the higher mass fraction of DMADDM the coating solution had, the lower metabolic activity and biomass accumulation the saliva-derived biofilms got, up to 10 mg/mL. In a previous study, DMADDM coating implant materials could inhibit the metabolic activity of a three-species biofilm [26], our results found that these anti-biofilm materials could also decrease the metabolic activity of microcosm biofilm which consist of decades of bacteria species. Besides, dental implant coating with DMADDM inhibits the extracellular matrix of saliva-derived biofilms, which embedded the bacteria in the 3D-biofilm and protected the bacteria from the antimicrobials [38,39,40]. However, there was no significant difference between 5 mg/mL and 10 mg/mL groups in anti-bacteria, indicating that 5 mg/mL DMADDM in coating solution could make the anti-bacterial ability of titanium implant reach the ideal.

In addition, the microbial ecosystem was sensitive to DMADDM. The coating solution with 1 mg/mL DMADDM was enough to make the microecosystem-regulation effect of modified titanium discs distinctly different from that of control group and reach the top. When the mass fraction of coating solution grew from 1 mg/mL to 5 mg/mL and 10 mg/mL, no extra difference in microbial community was detected. All of the dental implants coated with DMADDM solutions at various mass fractions could prevent the implant-related infections in an ‘ecological way’. *Actinomyces*, as an initial colonizer on titanium surface [13,41], was more abundant in the biofilms on titanium discs without DMADDM. Previous studies [7,9] have proved that the higher level of *Actinomyces* was associated with peri-implant disease. Another decreased genus in the biofilms on DMADDM modified titanium discs was *Neisseria*. It has been detected more abundantly on the titanium surfaces placed in periodontitis subjects [42] and patients suffering from periodontal disease [43]. However, DMADDM did not inhibit the growth of *Lactobacillus*, like what it did to *Actinomyces* and *Neisseria.* The relative abundance of *Lactobacillus* increased significantly in the biofilms on DMADDM modified titanium discs. It has been found that more *Lactobacillus* was detected in health populations in previous study [43]. Some other publications indicated that *Lactobacillus* was considered as the probiotics to prevent and treat periodontal disease and peri-implant disease via numerous clinical studies [44,45,46,47]. By reducing the host inflammatory cytokine response, it could improve the periodontal parameters [46,48]. Therefore, DMADDM modified dental implant inhibited the harm and enlarged the benefit, finally reshaping the microbial community of biofilm.

Polydopamine (PDA) coating enriched in catechol and amino groups, provides a surface for secondary reactions via Michael addition or Schiff base chemistry to create multifunctional coatings [49]. In this work, the AH-Ti disc is suitable for dopamine self-polymerized to form PDA coating and then the DMADDM was immobilized on titanium surface. This modification method improves the adhesion between titanium and coatings more effectively than the others, including sandblasting, calcium phosphate, coating with micro-arc oxidized titanium films, or antimicrobial peptide coating. The adhesion of DMADDM gave the short-term anti-bacterial ability to AH-Ti disc, which was proved by this study. However, the long-term effects need further study.

In conclusion, this study investigated the anti-bacterial and microecosystem-regulating effects of dental implant coated with DMADDM solutions at various mass fractions. The DMADDM modified dental implant not only inhibited the biomass accumulation and metabiotic activity of saliva-derived biofilm, but also regulated microbial ecosystem to healthier condition. It was found that 5 mg/mL DMADDM in coating solution could provide ideal anti-bacterial and microecosystem-regulating capabilities of titanium implants. This novel dental implant coated with DMADDM was promising in preventing peri-implant disease through inhibiting biofilms in an ‘ecological way’.

## 4. Materials and Methods

### 4.1. Coating the AH-Ti Substrates with DMADDM

The AH-Ti substrates was prepared according the previous publication [50]. The titanium discs were polished using 280, 320, 400, 600, 800, 1000 and 1200 grid sandpaper and sonication in acetone, ethanol, and ultrapure water for 30 min each and then air dried. The micro-nanostructure of titanium surfaces was prepared by alkali-heat (AH) treatment protocols.

DMADDM was synthesized via a modified Menschutkin reaction method as previously described [20] and immobilized on to AH-Ti substrates following the methods from Yang et al. [26]. In brief, DMADDM was dissolved in 50 mM Tris-buffer (pH 8.5) with a mass fraction of 1 mg/mL, 5 mg/mL and 10 mg/mL, respectively. Dopamine hydrochloride (Sigma-Aldrich, St. Louis, MO, USA) and hydroxyapatite were dissolved in the same buffer with a mass fraction of 5 mg/mL, which were used to assist the immobilization of DMADDM. Then the AH-treatment titanium discs were covered by 2 mL of the final solution in 12-well polystyrene plates, and stirred at 200 rpm for 24 h at room temperature. Finally, the AH-Ti Substrates were sterilized in an ethylene oxide sterilizer (Anprolene AN 74i, Andersen, Haw River, NC, USA).

### 4.2. Saliva Collection

The study was authorized by the Ethical Committee of Sichuan University (Chengdu, China). Seven healthy persons with natural dentition and without periodontal disease and active caries who did not take any antibiotics in the last three months were chosen as donors for saliva. The saliva from donors was pooled together and diluted two-fold with sterile 50% glycerol and was stored at −80 °C as described previously [51,52,53,54].

### 4.3. Biofilm Development

Each sterile AH-Ti disc was fitted into one well of a polystyrene 24-well flat-bottomed microtiter plate, containing 1.5 mL the SHI medium (see Appendix A for detail) [27]. The saliva-glycerol stock was seeded (1:30 final dilution) into the microtiter plates and incubated at 37 °C for 48 h anaerobically (90% N_2_, 5% CO_2_, 5% H_2_). The growth medium was refreshed every 12 h. Before immersing in the fresh medium, the saliva-derived biofilms were rinsed with phosphate-buffered saline (PBS) to remove loose bacteria [51,52].

### 4.4. Crystal Violet Assay and MTT Assay

To determine biomass accumulation, crystal violet assay was performed. The PBS-rinsed 48 h biofilms were placed into a 24-well plate. To be fixed, each biofilm was submerged in 1 mL 100% methyl alcohol for 15 min. Then biofilms on the disks were rinsed with PBS and transferred to a new 24-well plate, submerged in 1 mL 0.1% crystal violet solution for 5 min. To remove the residual dye, the biofilms were washed with PBS. Then, the disks were transferred to another 24-well plate. 2 mL 95% ethanol solution was added into each well and the plate was shaken horizontally at 80 rpm for 45 min at room temperature. 100 μL of ethanol solution from each well was diluted with 95% ethanol solution to 200 μL and transferred into 96-well plate. The microplate reader was used to measure the absorbance of the solution at the OD 595 nm.

MTT (3-(4,5-Dimethyl-thiazol-2-yl)-2,5-diphenyltetrazolium bromide) assay was used to measure the metabolic activity [19]. The PBS-rinsed 48 h biofilm on the disk was placed and 1 mL MTT dye (0.5 mg/mL MTT in PBS) was added into each well of the 24-well plate. Then these biofilms plate were cultured for 1 h (37 °C anaerobically). To dissolve the formazan crystals, the disks were fitted into a new 24-well plate filling 2 mL dimethyl sulfoxide (DMSO) and shaken horizontally at 80 rpm for 20 min in the dark. Finally, 200 μL of the DMSO solution containing the formazan crystals retained by the biofilms was pipetted into a 96-well plate to measure the absorbance at the OD 540 nm via the microplate reader.

### 4.5. Live/Dead Bacteria Staining

The PBS-rinsed 48 h biofilm on the disk stained using the BacLight live/dead bacterial viability kit (Molecular Probes, Eugene, OR, USA). Live bacteria cells were stained with SYTO 9 emitting green fluorescence, while dead cells were stained with propidium iodide emitting red fluorescence. Biofilms of each group were examined by confocal laser scanning microscopy (Leica, Wetzlar, Germany).

### 4.6. SEM Observation

For the scanning electron microscopy (SEM) examination, the PBS-rinsed biofilms on the disks were immersed in 1% glutaraldehyde for 4 h at 4 °C. Then, the discs were washed twice in sterile water (immersion time per washed, 10 min) and dehydrated via a series of graded ethanol solutions (50%, 60%, 70%, 80%, 90% and 100%; immersion time per series, 10 min), followed by sputter-coating with gold. Finally, scanning electron microscopy (SEM, Quanta 200, FEI, Hillsboro, OR, USA) was used to examine the biofilms.

### 4.7. 16S rRNA Gene Sequencing

The biofilms were subjected to Majorbio (Shanghai, China) where the total DNA was isolated, amplified and sequenced according to their standard procedures [34,55,56,57]. In brief, by the E.Z.N.A.^®^ Soil DNA Kit (Omega Bio-tek), DNA was extracted from the saliva-derived biofilms. Nanodrop (Thermo Scientific) and agarose gel electrophoresis were used to assess the DNA concentration and quality, respectively. 515F_907R barcoded primers were used to amplify the variable region 4 and 5 (V4–V5) of bacterial 16S rRNA by PCR, which were performed in a triplicate 20 μL mixture containing 4 μL of 5× FastPfu Buffer, 2 μL of 2.5 mM dNTPs, 0.8 μL of each primer (5 μM), 0.4 μL of FastPfu Polymerase, and 10 ng of template DNA. The amplicons were then extracted from 2% agarose gels and further purified by the AxyPrep DNA Gel Extraction Kit (Axygen Biosciences, Union, CA, USA) and quantified by QuantiFluor™-ST (Promega, Madison, WI, USA). On an Illumina MiSeq platform, purified amplicons were paired-end sequenced (2 × 300) according to the instructions. The raw data was uploaded to the NCBI Sequence Read Archive (SRA) database.

### 4.8. Bioinformatics and Statistical Analysis

Raw FASTQ files were demultiplexed and quality-filtered by QIIME (version 1.9.1) [58]. Based on the UPARSE (version 7.1), operational taxonomic units (OTUs) were clustered with 98.5% similarity cutoff. The taxonomy of each 16S rRNA gene sequence was analyzed by Ribosomal Database Project (RDP) Classifier [59] (http://rdp.cme.msu.edu/) against the Human Oral Microbiome Database (HOMD) with a confidence threshold of 70% [60]. Alpha diversity index (Simpson index) [61] calculations were performed on Mothur v.1.30.2. Phylogenetic beta diversity was determined based on the represented sequences of OTUs. Principal component analysis (PCA) was conducted according to the distance matrices determined by the represented sequences of OTUs for each sample.

Mann–Whitney U testing was performed to detect the significant effects of the variables at a *p* value of 0.05. SPSS21.0 (SPSS Inc., Chicago, IL, USA) software was used for the statistical analysis.

## Supplementary Materials

Supplementary Materials are available online. Figure S1 showed the relative abundance of *Porphyromonas.*

## Abbreviations

DMADDM	Dimethylaminododecyl methacrylate

## Appendix A

The SHI medium has the following composition: peptone, 10 g/L; trypticase peptone, 5.0 g/L; yeast extract, 5.0 g/L; KCl, 2.5 g/L; sucrose, 5 g/L; hemin, 5 mg/L; vitamin K, 1 mg/L; urea, 0.06 g/L; arginine, 0.174 g/L; mucin (type III, porcine, gastric), 2.5 g/L; sheep blood, 5%; and NAM, 10 mg/L.
