# Supplementary material for: Anti-Bacterial and Microecosystem-Regulating Effects of Dental Implant Coated with Dimethylaminododecyl Methacrylate
Source: Molecules. 2017 Nov 20;22(11):2013. doi: 10.3390/molecules22112013 (PMC6150392; doi:10.3390/molecules22112013)
Supplement: Supplementary file 1 [file molecules-22-02013-s001.pdf]

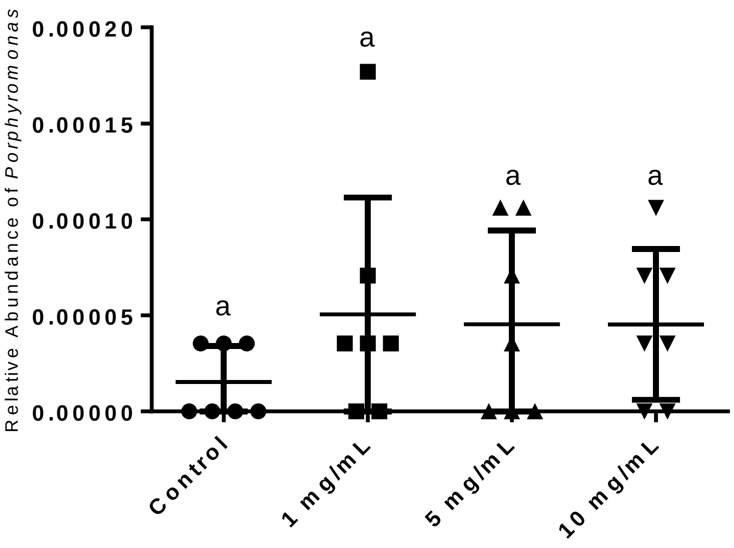

Figure S1. The relative abundance of *Porphyromonas*. Bars with the same letter indicate a value having no significant distance, and those with different letters indicate a significant difference ($p < 0.05$).
